# Lumbar vertebral canal stenosis due to marked bone overgrowth after routine hemilaminectomy in a dog

**DOI:** 10.1186/s13028-023-00700-2

**Published:** 2023-08-29

**Authors:** Francesca Tavola, Marco Ruggeri, Ines Carrera, Martí Pumarola, Pablo Menendez Alegria, Anna Tauro

**Affiliations:** 1 Awake Djursjukhus, Hornsgatan 150A, Stockholm, 117 28 Sweden; 2 ChesterGates Veterinary Specialists, Units E & F, Telford Court, Gates Lane, Chester, Cheshire, CH1 6LT UK; 3 Vet Oracle Teleneurology, CVS Limited, Owen Road, Diss, Norfolk, IP22 4ER UK; 4 Mouse and Comparative Pathology Unit, Department of Animal Medicine and Surgery, Veterinary Faculty, Networking Research Center on Bioengineering, Biomaterials and Nanomedicine (CIBER-BBN), Universitat Autònoma de Barcelona, Campus UAB, Bellaterra, 08193 Barcelona Spain

**Keywords:** Ataxia, Bone overgrowth, Inflammatory response, Laminectomy, Micromotion, Neurological deficits, Paralysis, Paraplegia, Spinal, Vasculopathy

## Abstract

**Background:**

Bone overgrowth after decompressive surgery for lumbar stenosis resulting in recurrence of neurological signs has not been reported in veterinary literature. However, there are few cases described in human medicine.

**Case presentation:**

A 13-month-old entire female dog, a crossbreed between a Springer Spaniel and a Border Collie, weighing 24 kg, was referred with a 5-day history of progressive spastic paraplegia, indicative of a T3-L3 myelopathy. Magnetic resonance (MR) imaging revealed a right-sided L2-L3 compressive extradural lesion, compatible with epidural haemorrhage, which was confirmed by histopathology. The lesion was approached via right-sided L2-L3 hemilaminectomy and was successfully removed. One-year postoperatively the dog re-presented with pelvic limb ataxia. MR and computed tomography (CT) images demonstrated excessive vertebral bone formation affecting the right articular processes, ventral aspect of the spinous process of L2-L3, and contiguous vertebral laminae, causing spinal cord compression. Revision surgery was performed, and histopathology revealed normal or reactive osseous tissue with a possible chondroid metaplasia and endochondral ossification, failing to identify a definitive reason for the bone overgrowth. Nine-month postoperatively, imaging studies showed a similar vertebral overgrowth, resulting in minimal spinal cord compression. The patient remained stable with mild proprioceptive ataxia up until the last follow-up 18 months post-revision surgery.

**Conclusion:**

This is the first report in the veterinary literature of bone overgrowth after lumbar hemilaminectomy which resulted in neurological deficits and required a revision decompressive surgery.

## Background

Bone healing after injury is a complex process involving various biological and biomechanical factors. The three phases of bone healing are the inflammatory phase, the repair phase, and the remodelling phase [1, 2]. Bone healing depends on the initial inflammatory phase, both local and systemic, in response to the injury. The repair phase is characterised by the formation of a soft callus, consisting of fibroblasts, chondrocytes, and osteoprogenitor cells. The new extracellular matrix produced by these cells serve as a temporary scaffold for bone formation. This phase involves the recruitment and differentiation of mesenchymal stromal cells (MSCs), which have the potential to differentiate into osteoblasts, promoting bone regeneration and bridging the fracture site. The remodelling phase is the final stage of bone healing, where the newly formed bone undergoes structural and functional modifications, restoring the bone’s normal architecture [3].

In human medicine, bone overgrowth is an uncommon but important cause of relapse of neurological signs after decompressive surgery in elderly patients undergoing treatment for lumbar spinal stenosis, most performed at the L4-L5 level [4–10]. The exact osteopathological mechanism underlying post-operative bone overgrowth remains unclear. However, factors such as segmental instability [8], micromotion [5, 6], and abnormalities in mechano-signalling have been hypothesised to contribute to the excessive bone regrowth [5]. Conservative management has been suggested as an initial approach to treating radicular pain caused by lumbar restenosis in human patients [11]. However, in many cases, repeated decompression surgeries are often required to address the bone overgrowth and alleviate clinical signs [5].

In the field of veterinary medicine, there is limited knowledge regarding vertebral bone regrowth after surgical interventions [12]. Furthermore, reports of vertebral bone overgrowth following spinal surgery are not found in veterinary literature.

We present a case involving a young dog which experienced lumbar bone overgrowth after a routine hemilaminectomy surgery. To the authors’ knowledge, this is the first report describing a severe bone overgrowth which required a revision decompressive hemilaminectomy.

## Case presentation

### Clinical presentation and diagnostic findings

A 13-month-old entire female dog, a crossbreed between a Springer Spaniel and a Border Collie, weighing 24 kg, was referred to ChesterGates Veterinary Specialists with a 5-day history of progressive paraplegia (Table 1). There was no history of trauma, however, the dog had been attending agility training prior to onset of neurological signs.

**Table 1:** Timeline of the clinical findings, diagnosis, investigations, and surgical intervention. CRP = C-Reactive Protein; CSF = cerebrospinal fluid; *n/a = not applicable; R = right; L = left*

TIMELINE	Clinical signs	Neurological signs	Diagnosis	Imaging	CRP (RI < 10)	CSF analysis	Surgical procedures
Day 0	Intermittent lethargy, hyporexia, GI signs	Spastic paraplegia with preserved nociception	L2-L3 epidural haemorrhage	MRI & CT	CRP = 64 mg/L	n/a	L2-L3 right sided hemilaminectomy
2 m after 1st surgery		Pelvic limb ataxia		n/a	n/a	n/a	n/a
1 year after 1st surgery	Intermittent lethargy and vomiting	Intermittent pelvic limb ataxia (R > L)	L2-L3 bone overgrowth	MRI & CT	CRP = 104 mg/L	n/a	L2-L3 right sided revision surgery
3 m after revision surgery	Occasional vocalisation	Pelvic limb ataxia		n/a	n/a	n/a	n/a
9 m after revision surgery		Pelvic limb ataxia	L2-L3 bone overgrowth	MRI & CT	CRP = 3 mg/L	Normal	n/a
18 m after revision surgery		Pelvic limb ataxia					

The dog was rescued from Romania at the age of 6 months and had a history of intermittent lethargy, hyporexia, and gastrointestinal signs. The patient had received regular vaccination and antiparasitic treatments.

Physical examination was within normal limits. Neurological examination revealed spastic paraplegia with preserved nociception and normal mentation. Postural reactions (paw placement and hopping response) were absent in the pelvic limbs, while myotatic reflexes were unremarkable. Thoracic limbs and cranial nerve assessment were unremarkable. There was no pain elicited upon spinal palpation. Overall, the examination was consistent with T3-L3 myelopathy.

Haematology and serum biochemistry profile revealed mild neutrophilia (13.9 × 10^9/L, reference interval (RI) 3.5–12). Coagulation tests, including prothrombin time (PT; 9.6 s, RI 14–20), activated partial thromboplastin time (aPTT; 89.8 s, RI 94–123), and buccal mucosal bleeding (BMBT; 4 min, RI < 4) were within normal limits. IDEXX AngioDetect™ test for *Angiostrongylus vasorum* and IDEXX SNAP 4Dx (Idexx Laboratories, Wetherby, UK) for *Dirofilaria immitis* antigen and *Borrelia burgdorferi*, *Ehrlichia ewingii*, *Anaplasma phagocytophilum*, and *Anaplasma platys* antibodies were negative. However, C-reactive protein (CRP) was elevated (64.0 mg/L, RI < 10), indicating the presence of systemic inflammation.

**Fig. 1:**
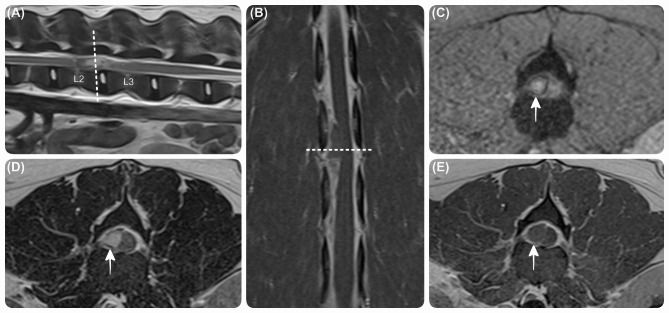
**Comparison between different magnetic resonance images at the level of L2-L3 vertebrae.** Sagittal T2W (**A**), dorsal T1W post-contrast (**B**), and transverse T2*GRE (**C**), T2W (**D**), T1W post-contrast (**E**) magnetic resonance images at the level of L2-L3 vertebrae, showing a right-sided extradural lesion (white arrow), extending from mid-L2 to mid-L3 vertebral body levels. The lesion showed heterogeneous, hyperintensity on T2W and T1W images, and had a complete hypointense rim and hyperintense centre on T2*-GRE images. The lesion exhibited faint peripheral contrast enhancement.

Magnetic resonance (MR) (Siemens MAGNETOM Essenza 1.5T) imaging of the thoracolumbar spine revealed a right-sided ill-defined extradural lesion, extending from mid-L2 to mid-L3 vertebral body levels, and causing severe compression of the spinal cord. The lesion was heterogeneous, hyperintense on both T2-weighted (T2W) and T1-weighted (T1W) images relative to the normal spinal cord. The T2*-gradient recalled echo (GRE) images showed a complete hypointense rim and hyperintense centre within the extradural lesion, with faint peripheral contrast enhancement (Fig. 1). Additionally, there was a poorly defined hyperintensity on the dorsal short tau inversion recovery (STIR) images within the epaxial muscle at the same level. All vertebrae were normal, and all visible intervertebral discs were well-hydrated without evidence of herniation. These findings were consistent with a right-sided epidural haemorrhage causing secondary spinal cord compression. The observed muscular changes were likely associated with secondary inflammation and/or oedema.

### Surgical intervention

The dog underwent a L2-L3 right-sided hemilaminectomy. A reddish, soft extradural lesion resembling a blood clot was found adherent to the dura mater, inner periosteum, and nerve root. The lesion was successfully removed, and its histopathological evaluation confirmed the diagnosis of epidural haemorrhage.

Following surgery, the dog’s condition gradually improved, showing moderate ambulatory paraparesis and pelvic limb proprioceptive ataxia. After a six-day of hospitalisation, the patient was discharged with a course of gabapentin to manage neuropathic pain (Actavis Ltd, Castleford, UK; 12 mg/kg, PO, q8h, for 2 weeks), diazepam to promote relaxation of skeletal muscles in the urethra and facilitate voiding (Teva Ltd, Runcorn, UK; 0.2 mg/kg, PO, q8h, for 1 week), and paracetamol/codeine for pain management (Pardale-V, Dechra Ltd, Northwich, UK; 8 mg/kg, PO, q8h, for 1 week). Considering the dog’s history of recurrent gastrointestinal signs, non-steroidal anti-inflammatory drugs (NSAIDs) were not used.

#### Follow-up and surgical revision

At two months after the initial presentation, the dog showed significant improvement, with only mild residual pelvic limb ataxia.

However, one year later, the dog was presented again at ChesterGates Veterinary Specialists with a history of intermittent lethargy, hyporexia, vomiting, and pelvic limb ataxia. Physical examination was normal. Neurological examination showed mild pelvic limb ataxia, worse on the right side, while the rest of the neurological examination was unremarkable. The neuroanatomical localisation was consistent with a T3-L3 myelopathy. Haematology, serum biochemistry profile, urinalysis (including urine culture), and ACTH stimulation test were normal. However, CRP was increased (104.0 mg/L, RI < 10).

**Fig. 2:**
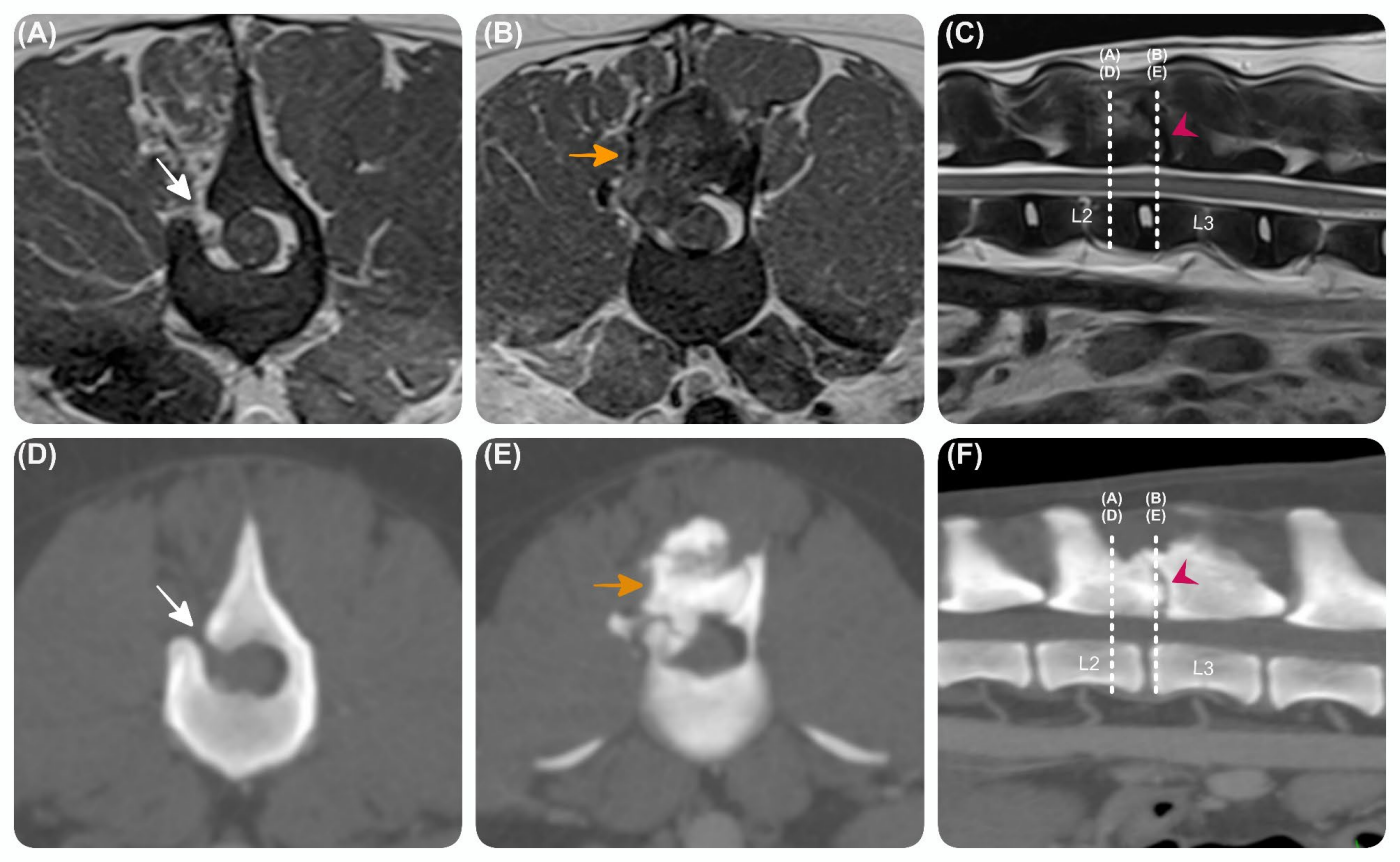
**Advanced diagnostic imaging performed a year post-routine hemilaminectomy.** Transverse T2W (**A**) (**B**), and sagittal T2W (**C**) magnetic resonance images and transverse (**D**) (**E**) and sagittal (**F**) computed tomography images at the level of the L2-L3 vertebrae, showing a relatively sharp and small laminar defect (H 1 mm x L 4.5 mm) (white arrow) on the right side of caudal aspect of L2 vertebra. Adjacent to this site and extending caudally, there was an ill-defined expansive lesion (yellow arrow) lesion arising from the vertebra, affecting the right articular processes, and ventral aspect of the spinous process of L2-3 and the contiguous vertebral laminae, causing mild spinal cord compression. The lesion was slightly heterogeneous in all sequences, with mild heterogeneous contrast enhancement.

Repeat MR study of the thoracolumbar spine revealed a small laminar defect (H 1 mm x L 4.5 mm) on the right side of the caudal aspect of the L2 vertebra, which was the site of the previous surgery. Adjacent to this site and extending caudally, there was an ill-defined lesion arising from the vertebra, affecting the right articular processes, the ventral aspect of the spinous process of L2-L3, and the contiguous vertebral laminae. This lesion was expansive in all directions, distorting the normal shape and joint space of the articular processes. It projected into the vertebral canal as an extradural lesion, causing mild spinal cord compression. The lesion appeared slightly heterogeneous but markedly hypointense in all sequences, with mild heterogeneous contrast enhancement. The signal intensity pattern indicated a likely mineralised nature (Fig. 2). In addition, there was a mild contrast enhancement of the adjacent meninges interpreted to be likely due to meningeal fibrosis, secondary to the previous surgery.

Computed tomography (CT) study of the entire spine confirmed the MR findings of a mineralised vertebral lesion. CT of thorax and abdomen did not identify any metastatic neoplastic disease.

The dog underwent a revision L2-L3 right-sided hemilaminectomy. However, the surgical procedure presented challenges due to the loss of normal anatomical landmarks caused by bone overgrowth. To ensure the accuracy of the surgical approach, a perioperative spinal CT scan was performed. The scan revealed that the initial surgical approach was too dorsal, and that the hemilaminectomy, performed using a pneumatic-powered drill, was initially targeting the spinous processes of L2-L3. To address this issue, a wider surgical approach was undertaken, and intra-operative fluoroscopy was used as a guiding tool. The remaining anatomical landmarks, including the accessory processes of L1-L2 and L3-L4, as well as the transverse process of the L2-L3 vertebrae, were identified. The L2-L3 right-sided hemilaminectomy was accomplished and revealed an oedematous spinal cord with a pinkish granulated appearance of the dura mater. The dorsolateral compression was evident and mainly affecting the L2 vertebra. Multiple samples, which had a mixed consistency of bone and fibrocartilage, were collected intraoperatively and sent for histopathological evaluation. Following the spinal cord decompression, a postoperative CT scan showed a minimal amount of residual bone overgrowth at the level of the rostral edge of L2, which did not cause significant spinal cord compression.

Following surgical procedure, the dog’s condition gradually improved, showing mild ambulatory paraparesis and pelvic limb ataxia. The patient was discharged three days after surgery. In addition to diazepam (Teva Ltd, RuncornUK; 0.2 mg/kg, PO, q8h, for 10 days), paracetamol / codeine (Pardale-V, Dechra Ltd, Northwich, UK; 8 mg/kg, PO, q8h, for 2 weeks), and gabapentin (Actavis Ltd, Castleford, UK; 12 mg/kg, PO, q8h, for 2 weeks), the patient received a tapering course of corticosteroids to reduce the local oedema and inflammation (Wockhardt Ltd, Wrexham, UK; prednisolone, 0.3 mg/kg, PO, q24h, for 10 days, then 0.2 mg/kg, PO, q24h, for 10 days, then 0.1 mg/kg, PO, q24h, for 10 days, the 0.1 mg/kg, PO, q48h, for 10 days) and pentoxifylline to improve microcirculation, reduce inflammation, and enhance the healing process (Trental, Sanofi Ltd, Reading, UK; 16 mg/kg, PO, q8h, for a month).

### Histopathological evaluation

The histological sections were stained with haematoxylin and eosin and scanned images were evaluated digitally. The submitted specimens consisted of histologically normal or reactive connective, adipose and osseous tissues with no evidence of active inflammatory, proliferative reaction, or neoplasia. In some fragments this reactive fibrous tissue surrounded fragments of thin osseous trabeculae of compact bone containing well organized osteons surrounding cavities filled with bone marrow. In other fragments, between a hyaline fibrillary matrix, multiple lacunae containing one or two cells mimicking isogenous group of chondrocytes were present. Some changes were supportive of regenerating activity of connective tissue (fibrosis). The absence of active inflammatory reaction could not rule out a possible chronic stage of the underlying process.

### Follow-up imaging and outcome

**Fig. 3:**
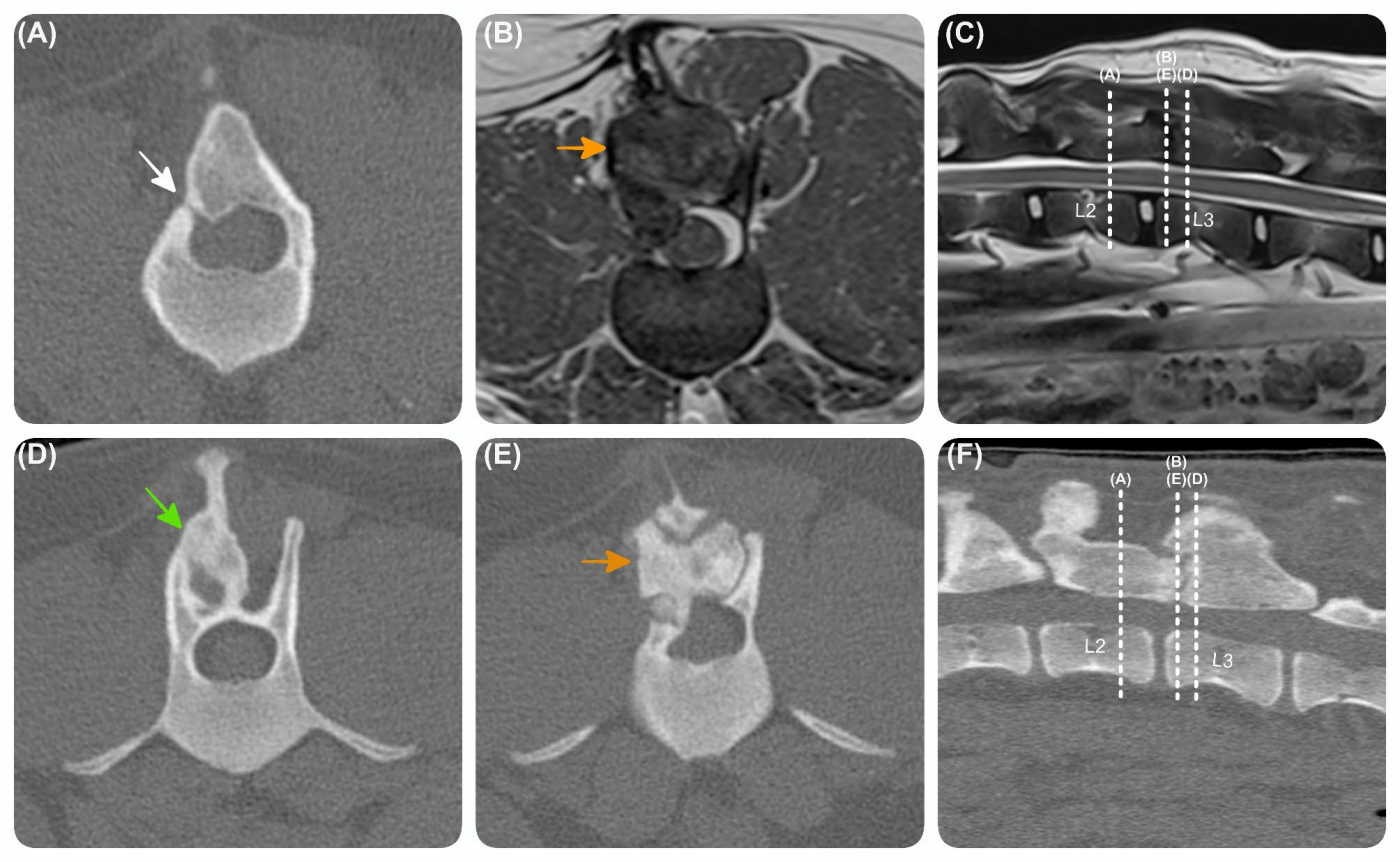
**Advanced diagnostic imaging performed 9 months post-revision surgery. **Transverse (**A**) (**D**) (**E**) and sagittal (**F**) computed tomography images and transverse T2W (**B**) and sagittal (**C**) magnetic resonance images at the level of the L2-L3 vertebrae, showing a right-sided expansive lesion (orange arrow) similar to the Fig. 2, with smooth bone almost completely filling the previous laminectomy defect, but leaving a small laminar gap (H 1 mm x L 1 mm), on the right side of the caudal aspect of L2 vertebra (white arrow). The lesion has grown more eccentrically, and incorporates the slightly deviated L2 and L3 (green arrow) spinous processes.

Three months after the revision surgery, the dog was presented with signs of occasional vocalisation suspected to be secondary to discomfort and showed slight pelvic limb ataxia. Additional monitoring was advised. No further neurological progression was noted, and the dog was re-admitted 6 months later (i.e., 9 months after the revision surgery) for repeating lumbar spinal MR and CT studies to investigate whether the dog’s occasional vocalisation was related to spinal pain and to assess the condition of the surgical site. CRP was now within normal limits (3 mg/L; RI < 10) and SNAP 4Dx test (Idexx Laboratories, Wetherby, UK) for vector-borne diseases was repeated and the result was negative. MR and CT studies of the thoracolumbar spine showed mineralised expansive lesion, similar in shape, margination and signal intensity compared to the pre-revision surgical images. The right vertebral lamina of L2 and L3 showed smooth bone almost completely filling the previous laminectomy defect, similar to the pre-operative state but with a smaller laminar gap (H 1 mm x L 1 mm), on the right side of caudal aspect of L2 vertebra. There was a noticeable bone growth in an eccentric manner, incorporating the slightly deviated L2 and L3 spinous processes. It is important to note that during the revision surgery, these spinous processes were mistakenly targeted (Fig. 3). Overall, the healing process had resulted in the vertebrae being reconstructed similarly to their pre-revision surgery state. Despite the presence of slight eccentric and concentric bone regrowth that extended beyond the normal vertebral margins, the resulting minimal spinal cord compression was not deemed significant enough to require another surgical intervention. No intramedullary lesions were observed, and the mild meningeal enhancement seen before was still faintly present.

Analysis and culture of the cerebrospinal fluid (CSF) collected via a lumbar puncture were normal (CSF protein 39.8 mg/dL, RI < 45; TNCC 1 cell/uL, RI < 5).

The patient was discharged with pentoxifylline (Trental, Sanofi Ltd, Reading, UK; 16 mg/kg, PO, q8h for a month) and paracetamol / codeine (Pardale-V, Dechra Ltd, Northwich, UK; 8 mg/kg, PO, q8h, PRN). Physiotherapy and hydrotherapy were recommended, and further monitoring of the clinical progress via video recording was advised. Approximately 18 months after the revision surgery, the patient was re-examined and found to be stable with mild proprioceptive ataxia of the pelvic limbs with no evidence of discomfort.

## Discussion and conclusions

The present case report is the first to document excessive bone overgrowth causing vertebral stenosis following lumbar spinal decompression in a canine patient.

While references to bone overgrowth as a surgical complication in lumbar stenosis exist in human literature [4–8, 13], there is a lack of specific veterinary literature addressing bony defect resolution following hemilaminectomy.

In human patients, post-surgical bone overgrowth typically evolves over several months to years, primarily in elderly patients over the age of 50. Various factors can influence this process [7, 13], such as post-operative vertebral instability [8] or micromotion [5]. This is commonly observed at the L4-L5 level, known to be the most mobile intervertebral level in humans [4–8]. Excessive motion or inadequate mechanical environment can disrupt normal bone remodelling, leading to cartilage and bone formation, as well as an excessive inflammatory response to the initial trauma [5, 14]. This can subsequently lead to abnormal bone growth. It is important to note that systemic abnormalities are not typically apparent in these patients [5].

In contrast to the human literature [4–10], our case report describes excessive bone overgrowth following a hemilaminectomy procedure performed in a young dog to remove an extradural hematoma. It is worth noting that the affected spinal level in our case is not typically considered the most mobile segment in dogs. Although overt signs of instability were not observed, a dynamic study was not conducted to confirm the absence of instability [5], [6]. Vertebral stabilization was not performed in our canine patient, as we believe that a different pathophysiological mechanism may be responsible for the observed bone overgrowth. We hypothesised that an excessive inflammatory response triggered by the surgical insult could have contributed to the post-surgical bone overgrowth. The initial presentation of our patient with an extradural haematoma and increased CRP level may supports the presence of an excessive systemic inflammatory response. Additionally, the history of intermittent gastrointestinal signs may further suggest an abnormal immune response; although, this could not be confirmed. The main limitation of our report includes the performance of CSF analysis only 9 months after the revision surgery, which may not have captured the complete picture of the inflammatory response during the initial stages of the condition. Additionally, we acknowledge the absence of immunoglobulin A (IgA) concentration testing for canine steroid-responsive meningitis-arteritis (SRMA) [15]. However, the resolution of the clinical signs without the use of corticosteroids at immune-suppressive doses, along with the absence of typical clinical signs such as pyrexia and spinal pain [15], make the diagnosis of canine steroid-responsive meningitis-arteritis (SRMA) less likely.

In our case, we opted for a course of anti-inflammatory dose of corticosteroids and pentoxifylline in an attempt to reduce the suspected inflammation and vasculopathy. Although the definitive benefit of these drugs in our patient remains uncertain, we did not observe any side effects associated with their use.

In humans, a revision surgery is often required to treat restenosis [5], and this approach was chosen for our canine patient, which also allowed for a histopathological evaluation of the bone overgrowth.

Histopathological evaluation showed the presence of normal or reactive osseous tissue, rather than new bone and immature woven bone. This finding may be similar to heterotopic ossification reported in humans following trauma or injury. The presence of chondrocytes in some fragments could indicate a possible chondroid metaplasia and endochondral ossification, which are necessary for bone regrowth [5, 16]. Despite thorough histological examination, we did not identify a definitive histological reason for the bone overgrowth, in our case. However, it may be also that the bone biopsies, although obtained from different sites, may not have been fully representative of the entire lesion. It is possible that the underlying mechanism of bone overgrowth in our canine patient involves complex cellular interactions and tissue remodelling processes that were not captured in the biopsied samples. Understanding the complex cellular and molecular processes involved in bone healing is essential to better comprehend the underlying mechanisms of excessive bone overgrowth observed in certain cases.

In conclusion, this case report is the first to document bone overgrowth following lumbar decompression surgery in a canine patient.

## Data Availability

The datasets used and/or analysed during the current study are available from the corresponding author on reasonable request.

## Declarations

### Prior publications

Data have not been published previously.

## Abbreviations

CSF: Cerebrospinal fluid
CRP: C-reactive protein
CT: Computed tomography
GRE: Gradient recalled echo
L: Lumbar
MR: Magnetic resonance
MSCs: Mesenchymal stromal cells
RI: Reference interval
SRMA: Steroid-responsive arteritis-meningitis
STIR: Short tau inversion recovery
T1W: T1-weighted
T2W: T2-weighted
TNCC: Total nucleated cell count
